# Hysteria

**Published:** 1880-07

**Authors:** J. A. Goldsberry


### ARTICLE III.

HYSTERIA. By J. A. GOLDSBERRY, M. D. Read before the Parke County, Ind., Medical Society.

Hysteria is a malady whose origin doubtless antedates by many hundred years, the first case of which history or science furnishes any record; indeed I believe it to be coeval with the human race, and therefore if I would seek for the individual in whom hysterical phenomena first had a beginning, I would go back to "the mother of all living," to the person of Eve herself; for I doubt not that in that memorable period in her history when "she heard the voice of the Lord God walking in the garden," standing in His immediate presence she made that sad confession,—"the serpent beguiled me and I did eat," and this ackowledgment immediately followed by the Divine anathema, "I will greatly multiply thy sorrow and thy conception; in sorrow thou shalt bring forth children, and thy desire shall be to thy husband, and he shall rule over thee." While these events were transpiring, or, it may be, very soon thereafter, I repeat, I doubt not, amid circumstances, which, to her, must have been thrillingly exciting—that Eve's emotional nature was profoundly impressed, so much so, that for aught we know, for the first time in her life, she betrayed one of the weaknesses of her sex. and exhibited in her own case, perhaps in a striking manner, many of the spmptoms characteristic of the disease under consideration. Is the hypothesis unreasonable? if so, why? She had been guilty of a grievous sin, for which she had incurred the Divine displeasure: not only so, but because of her act of disobedience she had entailed upon herself and all who were to come after her, the consequences of that sin, which were disease and death: a fact of which she had full knowledge. She now possessed, to their fullest extent, all of the frailties of weak human nature: she was subject to disease and the aches and pains connected therewith, both physical and mental, and was already, doubtless, in bitter grief, remorse and humiliation, reaping the fruit of her own sowing. Indeed it is difficult to conceive of circumstances, which, in their nature, were better calculated to develop the manifestations peculiar to this affection, than those surrounding this Bible character at the particular time of which we speak. The necessary factors were all present. I am forced, therefore, to the conclusion that our maternal first-parent may have been the victim of hysteria. But lest I be considered a little speculative I will address myself to a consideration of the disease of more recent date—of to day, and with your indulgence, endeavor to adduce a few thoughts relating to this singular ailment. Hysteria is a disease peculiar to females. Why this should be so, is a problem, which, I think, will not admit of very clear solution. The doctrine of some of the early fathers in medicine regarding this affection was crude and ludicrous. They located the disorder in the uterus, and attributed many of the morbid phenomena to the "wanderings" of that organ from one part of the body to another. The muscular movements, in whatever parts these might be, the *globus hystericus*, were all due to the immediate presence of the womb. Another belief was, I think a much more rational one, that the disease originated in the brain itself. Hence we read of "cerebral hysteria," as we do also of "uterine hysteria." Since the days of Hippocrates, hysteria has been a constant source of vexation, and not a little mortification to the medical world; not because of its danger and fatality, for unfortunately, if you will pardon the expression, for many of those who are its victims, it never destroys life; I repeat that it is not because of its danger to life that the profession fear an encounter with it—that the medical man approaches a case of the disease with so much reluctance—but by reason of the generally acknowledged fact, that science with all of her many grand and brilliant achievements in the field of medicine, tells us, as yet, but little of the nature and pathology of this very common affection. That hysteria is a species of the neuroses; that it is primarily a disease of the cerebro-spinal center; that somewhere within that wonderfully complex system, the germ is to be found from which spring the varying and varied manifestations which characterize the disorder, is a conclusion, I think, to which we must all come at last. In a disease whose occurence is so frequent, and with whose symptoms the gentlemen before me are doubtless more conversant than myself, it would seem unnecessary that I consume your time in dwelling upon its clinical history, but as a paper of this character could not be considered complete without making some reference to that division of the subject, with your permission I will speak briefly in a rather general way, of some of the more prominent symptoms, after which I will submit a report of two or three cases which came under my personal knowledge and management; will also in conclusion make brief mention of the pathology, etiology and treatment of the disease. In this, as in all other ills to which flesh is heir, the morbid manifestations vary greatly in different individuals, and in no other ailment, perhaps, more so than in the one in question. The abnormal disturbances in one case may present scarcely a single feature in common with those of another. Not unfrequently the only observable symptom of illness is a loss of voice, coming on suddenly, and lasting, it may be, for months, the power to speak, without any apparent cause, returning as suddenly as it was lost and the case is well.

Again, there is exaggerated emotional disturbance affecting mental action in various ways; the patient may be laboring under a simple depression of spirits, or she may have a violent fit of weeping, or she may cry and laugh alternately. In another case the morbid manifestations are violent in the extreme, the patient screams at the top of her voice, sheds tears profusely, becomes very voluble when she is suddenly seized with convulsive movements, which may be local or general. In another class of cases the only departure from the normal state consists in some impairment of motility, which differs greatly in different cases, as to its completeness, character, and extent. In one we may have paralysis in the form of paraplegia, in another hemiplegia; or the part affected may be one or both hands, or even a single finger. There may be paralysis of some particular group of muscles, or what is more singular, a single muscle. We have recorded examples of the latter in the levator palpebræ superioris, and in one of the recti of the eye ball.

The laryngeal muscles are sometimes involved, or œsophageal, giving rise, when in the former, to loss of voice or aphonia, which may be complete or incomplete, when in the latter, difficult and imperfect deglutition. Or the paralysis may be, apparently, general, affecting every voluntary muscle in the body. Again, there may be muscular spasms, involving, as in paralysis, a single muscle, or a particular set of muscles. The spasms are more frequently clonic in their character, but may be tonic, and when the latter, may last an indefinite period. The functions of the internal organs are always more or less impaired, thoracic and abdominal, but more uniformly the latter; the disturbances may be of the character of gastralgia or enteralgia, indigestion or obstinate constipation; pain of a neuralgic character in the uterus, ovaries, or bladder. When in the latter situation, the disorder is likely to be rebellious to all methods of treatment and exceedingly protracted, as I can testify from personal knowledge obtained from a long siege of perplexing experience in the management of a case thus affected. In still another class of cases the disease is characterized by deranged sensibility. This may be greatly exaggerated, constituting *hyperæsthesia*, or there may be a loss of sensation, resulting in complete *anæsthesia*. The different organs or parts of the body which may be involved in this morbid condition, of course, vary with different individuals. In one we may have hyperæsthesia of the integument, local or otherwise; in another it is confined to the muscles, and when the latter, more frequently those of the abdominal and thoracic regions than elsewhere.

Headache is a very common trouble, and among all the aches and pains and the almost innumerable morbid manifestations which characterize hysteria, no single one of them, I have thought, was more uniformly and constantly present than this one. Some one or more of the joints may be affected, simulating articular rheumatism. The internal organs are all more or less likewise involved; indeed, there does not seem to be any part of the body, internal or external, which is exempt from the impress of this particular form of hysteria. Loss of sensibility, or anæsthesia, though not so frequent in occerrence as the condition just alluded to, is, in a case now and then, a prominent symptom. While any part of the body may become anæsthetic, the skin is usually the seat of the disorder. A distinguished writer* on nervous pathology says: "In the days of witchcraft many an hysteri-

* Wm. A. Hammond.

cal woman, with anæsthetic spots on her skin, went to the gallows or the stake on suspicion of being leagued with the devil. The belief was that whenever the hand of the arch fiend or his assistants touched the skin, the spot at once lost its sensibility." It is not expected that in any given case of hysteria there must be present any great number of the almost innumerable list of symptoms which belong to the disorder. On the contrary, there are individuals laboring under the disease who present no other morbid manifestation, perhaps, than an obstinate headache or paroxysmal gastralgia, or cutaneous hyperæsthesia, or anæsthesia, it may be a tonic spasm, with contraction of a finger; yet these cases are none the less hysterical than are those in whom the symptoms are numerous, well marked, and highly characteristic. In both the former and latter all of the morbid phenomena depend upon the same primary cause, all have a common origin, and all possess a common nature. Following is the clinical history of three cases, the first of which, for obvious reasons, I give somewhat at length. I was called on the morning of Aug. 9, 1874, to visit Miss D., whom I found, as she remarked, "feeling badly." She was a young lady of intelligence and culture, of sanguine temperament, rather full habit, and twenty years of age. She usually enjoyed fair health, but now and then, was subject to attacks of what seemed to be neuralgia of the stomach, for the relief of which I had frequently given her professional attention. She was lively and vivacious in disposition, with a nature in which the emotional element was largely predominant; was a professor of religion, and the purity of her life was questioned by none. She was the daughter of wealthy parents, her home was one of peace and plenty, and she was surrounded with everything, it would seem, calculated to make life comfortable and happy. On making inquiry as to what she meant by the expression "feeling badly," she replied that while she was not particularly sick and was comparatively free from pain, there was a terrible, indescribable sensation in the region of the stomach, of which she must be relieved without delay, otherwise she should certainly die. A careful examination of the case lead me to believe that my patient's conclusions as to her real condition were not well founded. Save a slight indigestion, with constipated bowels, there was, apparently, no cause for what she insisted was a serious attack of illness; certainly there was nothing in her physical condition calculated to excite, in my own mind, the least anxiety. There was, however, a morbid mental state, evinced by gloomy forebodings and a marked depression of spirits. On the second day I found her very much the same; she had not slept well through the night; was rather less dejected, however, was more talkative and would occasionally smile; still complained very much at the epigastrium.

From a cathartic given on the first day her bowels had been freely moved; would take her meals regularly but had no relish for food. On the morning of the third day the true nature of the disorder was more fully defined; there was now, from the slightest pressure, apparently, great pain in the epigastric region. She complained of a feeling of fullness or choking sensation in the throat, the mammary glands were highly sensitive, as was also the integument covering the face. The muscles of the eye-lids were more or less convulsed. She was at times very talkative; would weep and shed tears profusely one moment, as one laboring under some deep mental anguish, and the next she would break forth in hearty laughter. She had no inclination to sit up even for a single moment. She was sensitive to any sudden noise, and seemed to desire the seclusion of a darkened room. On the evening of the same day I was called in great haste, found the family in the utmost confusion and alarm. A little while before, the patient, while engaged in quiet conversation with the mother, was suddenly seized with a violent convulsion.

When I reached her bed-side she was in the midst of a second paroxysm. The whole body was in a frightful state of agitation, and, contrary to the rule in such cases, the muscles of not only the trunk and extremities, but those of the face also, seemed to be involved in the convulsive movements. Her body was jerked and thrown in many different shapes, flouncing from one side of the bed to the other, sometimes assuming a position which was amusing if not ludicrous. An attempt to restrain her would apparently intensify the violence of the paroxysm; if a hand was being held she would free it if possible and immediately afterwards, would not infrequently, seize hold of her hair and pull with such force and persistency as to almost completely double the body. Occasionally there would be movements not unlike those observed in chorea. One leg would be drawn up with great rapidity and force, and again quickly extended, followed immediately by a like movement in the other leg. Spasm of the pharyngeal muscles was a very prominent, and, to the patient, certainly a very distressing symptom. She would frequently grasp the throat with one or both hands as if there was something there that was choking her to death, and would frequently not release her hold until the spasm had relaxed, when her hands would drop and she would utter a low, plaintive peculiar wail which was anything but pleasant to the ears of the bystanders. There was hyperæsthesia, apparently, over the entire body; the slightest touch of the surface, accidental or otherwise, was immediately followed by a sharp cry or sudden movement of the body, indicating a degree of exaggerated sensibility very unusual. The eyes, while exhibiting the phenomena so common in this affection, were more profoundly affected than in any case I had ever witnessed; the entire orbital group of muscles of either side seemed greatly agitated, so much so that the eyeballs were in constant and rapid movement, dancing and jumping about in a very strange and excited matter, to which, when added the clonic spasms of the eyelids, with incessant nictitation or winking, together with more or less contortion of the facial muscles and a rather demoralized condition of her toilette, and there was presented in the physiognomy of this otherwise sensitive and refined female a not very agreeable or pleasing aspect. Strangely enough, during all of this violent perturbation there was very little disturbance of the circulation, the pulse maintaining throughout very nearly its normal character, a fact however which obtains in many similar cases of this disease. While volition was seriously impaired, it was by no means altogether lost, as there were unmistakable evidences of partial consciousness. This fact was plainly observed during the paroxysm, as two or three times while in the height of the spasm, she would have fallen from the bed had she not, to avoid the mishap, voluntarily changed the position of the body, a circumstance showing that the muscular movements were not wholly automatic. The paroxysm lasted half an hour or more, and the patient signalized the closing finale by a copious discharge of urine which pretty thoroughly saturated her own linen and the bedding as well. Soon afterwards she fell into a tranquil sleep which continued a couple of hours, when, on waking there were again strong convulsive symptoms, which were soon controlled by an anæsthetic.

During the greater part of the fourth day she was free from convulsive attacks, refused to take food or medicine, was easily excited, would now and then weep in a rather quiet way, was not inclined to talk and seemed exceedingly sad and dejected. Late in the evening of the same day she had two paroxysms; these were not so protracted and were less severe than the one of the preceding evening, but as regards the morbid manifestations, did not differ materially. On the fifth day her condition was worse. She had not rested well through the night. In the morning she had two or three light paroxysms and the mental faculties were greatly excited. She would talk, laugh and cry, alternately, and made use of many expressions that were very foolish and absurd. She still refused to take medicine, and could not be prevailed on to taste food. She would occasionally drink a little water, but seemed to swallow with the utmost difficulty. On the sixth day she was no better; while there were no convulsive seizures, mental action was greatly disordered. The faculty or power of perception seemed to be almost completely abolished. Her mind was therefore rich in illusions and hallucinations. Her mother's voice, in an adjoining room, she insisted was that of an individual whom she had never met but once. The familiar bark of a harmless old dog was mistaken for that of a wild animal that she had never seen or heard. Noise produced by the slamming or sudden closing of a door was taken for the report of a pistol, and many of the sounds in and about the house which salute the ears a dozen times a day she attributed to causes altogether foreign to those producing them. She would declare, with great vehemence, that there were persons standing before her who, at the time, were many miles away. Again, she would see a certain savage animal, the size, shape and color of which she described with singular accuracy. She heard sounds of many kinds and of almost every quality and character, when all about the house, save herself. was as silent as the grave. On the seventh day she was very much the same. She was still busy in looking at imaginary objects, in listening at imaginary sounds and in smelling imaginary odors. She was induced to take a little food, also a dose or two of medicine On the eighth day she seemed better. Under the influence of chloral hydrate she slept during most of the night. She was calm and quiet, and the almost innumerable illusions, which for three days and nights had been holding high carnival with the mental forces, had all disappeared. She would take food when requested, but had no relish for it. She complained of headache, confined to the supra-orbital and frontal regions, also a slight gastric disturbance. Sensibility, cutaneous and muscular, appeared to be very nearly normal. She was not inclined to converse much, but when induced to talk she did so intelligently. While, as just observed, she was apparently better, there was an air of gloom and sadness in her general demeanor, which, I was fearful, might be the precursor of another outbreak, or if it were possible, some new development in the case. The sequel proved that my suspicions were well founded. On the morning of the ninth day I received a very pressing summons to visit my patient without delay. On repairing to the house I found, sure enough that the drama presented a new character. Early in the morning, soon after a violent fit of weeping, she passed into a deep sleep, from which it had been impossible to arouse her. Up to this date, as strange as it may appear, the family, particularly the mother, seemed wholly in the dark as to the nature of her illness, and hence had experienced no little unnecessary alarm and anxiety. Her mother was at the bedside crying bitterly, in the belief that her daughter was dying with, as she termed it, "brain fever." On carefully examining the case, it at once became evident, to my mind at least, that I now had before me a genuine case of *hysterical coma.* I assured the mother in a very positive manner, that she was greatly mistaken in her conclusions; that her daughter was not dying and was not likely to die very soon; that the disease under which she was laboring was void of danger and that her ultimate recovery was a matter of almost absolute certainty. But she was incredible, continued disconsolate and expressed great surprise that I should thus try to deceive her. The patient lay in the supine position with the legs extended, and so far as I could discover, there was not the least movement of any part of the body. To look at her, she appeared like one in a natural sleep. Her eyes and mouth were closed and the respirations were normal, except that they were remarkably quiet and easy. The pulse, though rather feeble, did not exceed 80 beats in the minute. A strong effort to arouse her, during which I resorted to various and many expedients, failed most utterly; the coma seemed genuine and complete, and after all, it was not surprising that the friends looked upon the new development as one of serious import. On the tenth day she was no better; in some respects she appeared worse. She was still sleeping profoundly, and had not moved her body, so far as noticed, for a period of thirty-six hours. Sensibility, both special and general, was apparently abolished. The integument was pricked and pinched, the vapor of ammonia was applied to the nostrils, loud and startling sounds were made near the ears, the eyelids were raised and the conjunctiva manipulated in such a way as would ordinarily have caused the severest pain, but amid all of this torture she lay as one dead; there was not observed the movement of a muscle. The eyelids were constantly closed, and on separating them they would gradually resume their former position; the pupils were a little contracted, and responded slowly to a bright light. The pulse, temperature and respiration were normal. The mouth was easily opened, but there was no effort at deglutition, as fluids introduced into the mouth were allowed to remain or flow out. There was not the slightest rigidity of the cervical or dorsal muscles, nor of the extremities. Indeed, the voluntary system of muscles, as regards sensation and motility, was practically so much dead matter, whose connection with the cerebro-spinal center had ceased to exist. The abdomen was tympanitic, a condition which had not been observed before. The bowels were constipated and were moved with difficulty; nor had she urinated for forty-eight hours. As the bladder was very much distended, I decided to use the catheter, which was done without the least resistance on her part, and apparently without her knowledge.

This case had now been on my hands for a period of ten days and such had been the anxiety and consequent importunity of her friends, that I was almost *forced* to spend a good part of my time at her bedside. The medical treatment thus far had been without any beneficial results: indeed her condition seemed less hopeful than at any time previous. While, it is true, the case has been, in many respects, an unusual one, at least as regards her persistent refusal to take almost everything that was offered her, dietetic and medicinal, thus seriously embarassing all efforts in that direction, it occured to me, for reasons which are entirely satisfactory to myself, that if she was cured in any reasonable length of time, the institution of a radical change in the method of treatment would become necessary. To this end, as an initiatory step, on the evening of this day I applied to the epigastrium a large blistering plaster. This procedure met with the ready concurrence of the family. As has been already observed, the patient had had occasional attacks of gastralgia, which, her mother thought, might be a factor for evil in the present attack of illness.

Before leaving the room, speaking so that all present might fully understand me, I stated most positively that my main object in blistering was to arouse the patient, that I would return early the following morning and if the desired result had not been secured, I should then blister the back of the neck, the temples and perhaps the whole length of the spinal column. I had entrusted to the confidence of a friend, a lady of intelligence and prudence, the programme decided upon, with my reasons therefor, and requested that she remain with the patient through the night, which she consented to do. I gave her full instructions as to the management of and the nature of the intelligence she should convey to the patient, should she awake before my return the next morning. About five o'clock the following morning, for the first time in forty-eight hours, she was noticed to move the body slightly; this was directly followed by her drawing up one leg, then the other; soon after which she rolled slowly over on the right side. Presently she opened her eyes, looked about the room, and, on being asked how she felt, replied that she was very tired. While she was inclined to be somewhat melancholy, she was evidently free from any mental aberration. She ate some with relish and soon expressed a desire for more. My friend above alluded to, regarding this as a favorable opportunity, choosing a time when no one was present, delivered to the patient the message, the substance of which was that for more than a week she had been a source, to her parents, of a great deal of unnecessary trouble and alarm, from the belief on their part that she was seriously ill; that her case was simply one of pure hysteria, and consequently, as she herself was aware, quite void of danger. That it was a disease, which, in a great measure, was under the control of the will. Her case was therefore virtually in her own hands; if she chose to exercise a little self-control and will-power, her cure would be sure and speedy, otherwise she would lie there indefinitely. When she was informed that this information was given to her by my authority and request, she manifested great surprise that I should treat her in such a way, and declared that my action was not only ungentlemanly, but unpardonable; presently she began crying and seemed much affected, insisting that I was bitterly cruel and unjust. In a few minutes she ceased her crying, became apparently indignant, rose up, called for her clothing, dressed herself and fairly sprang from the bed, remarking, if she was not sick there was no use in her lying there any longer. The following day she was out driving, *cured.* So much for a little moral therapeutics. So far as could be ascertained, the only cause of the attack in this young lady, the history of whose case I have imperfectly given, was disappointment in a love affair. She subsequently married, has since borne two or three children, and, I think, has had no return of the hysteria.

CASE II. This case was that of a married lady, aged 25 and the wife of a farmer. About four months subsequent to her first confinement she was taken, during the night, with an oppression, as she expressed it, in the region of the stomach; this was soon followed by a feeling of fullness of the throat, characterized by more or less dyspnœa. I saw her the following morning in connection with my friend Dr. Ira Gillum. She complained of a sense of fullness at the epigastrium and upper bowels. There was evidently a highly flatulent condition of the stomach, evidenced by frequent gaseous eructations. She had pain in the top of the head, also in the supra-orbital region. There were no evidences of any uterine trouble save a slight degree of retro-flexion. There were no convulsive attacks, nor had there been any, but there was some mental aberration, characterized mainly by the morbid interest she manifested in her own case and the many absurd reasons she gave for the development of this or that symptom. She claimed not to sleep well, and, when asleep, was annoyed with all kinds of unpleasant and frightful dreams. Her pulse was normal, appetite fair, bowels inclined to constipation. She remained very much in the condition above described, now a little better, then worse, for three months, when she suddenly became paraplegic. She attributed her inability to walk to prolapsus uteri. When informed that she was mistaken, that there was no prolapsus, she declared that the womb had escaped from the vagina and was plainly visible. No amount of persuasion could induce her to make the least attempt to walk unaided. The paralysis did not involve the sensory nerves nor was motion entirely lost, as, with help she would now and then walk a little, but in doing so her feet were scarcely lifted from the floor, her steps being made by a dragging or sliding movement. She would sit in a chair for hours, but would always have to be carried to it, and while sitting up would not unfrequently employ her time in reading, needle work, etc. Her appetite was good and she indulged it to the fullest extent, and save an irregularity of the bowels, due, doubtless, to a want of exercise, she was in excellent general health.

About four weeks subsequent to the attack of paraphlegia, she was suddenly seized with a desire to visit a neighbor's one-half mile distant. Her paralysis disappeared as if by magic, and she made the round trip unaided and with comparatively little fatigue to herself, but she had hardly reached her home when the paraplegia returned. Four more weeks elapsed, during all of which time, she refused to walk, insisting, when interrogated in regard to her condition, with apparent earnestness and candor, that the source of the whole trouble was the prolapsed uterus. Wearied and vexed beyond endurance, I concluded that I would try the experiment of becoming a convert to the opinion of my patient as to the cause of her helpless condition. This was a happy thought and delighted her beyond measure. I assured her that the difficulty could be relieved, and explained to her the method of treatment which would be necessary, to which she readily assented. This consisted in simply introducing into the vagina one of Hodge's lever pessaries. While, as already stated, there was no prolapsus in the case, nor had there been any, the use of the instrument had the desired effect. It righted the displaced organ and the restoration of motility followed as a natural consequence. The pessary had not been worn but a few days until she was able to walk with comparative ease. At least two months have elapsed since the paralysis was relieved, and while it cannot be said that she is not laboring, to some extent, under the hysterical condition, there has been no return of the troubles just referred to.

CASE III. This case was that of a young married woman, 26 years of age, of fine physical development, who had for years enjoyed uninterrupted good health. She was of a highly nervous temperament, and was irritable and obstinate in disposition. She was the second wife of her husband, who had a family of several children. With these, on one occasion, she became greatly exasperated, so much so that she was quite uncontrollable. Soon after this paroxysm of ill temper, she had a fit of crying, then she took to her bed, became quiet, and for three days scarcely spoke to any one. During this time she ate regularly, was conscious and did not complain. On the fourth day she closed her eyes, and claimed that it was impossible for her to open them. On the fifth day I was called to see her for the first time. She was lying on the bed, resting comfortably, was entirely free from pain, also from any undue excitement. She was rational and conversed quite freely; indeed her blindness, as she termed it, seemed to be the only feature in the case that was clearly abnormal. Having, as yet, received no information relating to the case, I was inclined at first to attribute the difficulty to a paralysis of the third pair of nerves, but as this was not likely to occur in the absence of symptoms pointing to cerebral lesion, and no such symptoms were observable—this fact, together with what I was able to learn of the circumstances immediately connected with the beginning of her ailment, led me to the conclusion that the case was one of hysteria. On raising the lids, which was easily done, the balls were found rolled upwards, so much so, that it was impossible to get a view of the pupils. For ten days the condition of the patient remained very much the same. Her general health was good, but the local disorder continued. At length I concluded I would try the virtue of blisters about the head and neck. To this she entered an emphatic protest and requested that I wait another day. I returned the following morning and found her sitting up, *with eyes open*, which in appearance were quite normal. She was lively and cheerful, and expressed herself as feeling unusually well. Her household duties were at once resumed and so far as I know she has had no return of the malady.

As to the cause of hysteria, but little seems to be absolutely known, and in this regard, with this, as with many other disorders that afflict humanity, the profession is not altogether a unit. While some, distinguished in the field of pathology, look upon it as an idiopathic disease, having its origin in some or all of the nerve centers, and of course wholly independent of reflex influence; others, scarcely less eminent, are of the opinion that it is primarily a local disease, and that its seat may always be traced to the sexual organs. In other words, that hysteria is a "reflex nervous derangement due to sexual irritation."

It is said to be a disease peculiar to females, but that it sometimes occurs in the male, there is no doubt, as quite a number of well authenticated cases of the latter have been reported. Within a recent period a well-defined case of the disease in the male was under my own charge. The patient was a young man, who admitted that he had been for years addicted to masturbation, which, doubtless, in this case, was the chief factor in the production of the disease. There can be no question, however, that sex stands at the head of the list among the predisposing causes of hysteria. Among others may be mentioned age, heredity, and the civil condition as it relates to the married or single state. Of those who are attacked with it, the vast majority are between the ages of 15 and 25; and as to hereditary influence, if the physician will take the pains to inquire into the antecedents of cases of hysteria which may from time to time come under his care and observation, he will find that a large proportion of them "had either hysterical mothers, aunts, or grandmothers." The condition of celibacy unquestionably predisposes to the disease. Why it should do so, is a question, in regard to which there are, of course, a variety of opinions.

Of exciting causes, their name is legion. Any circumstance or event, whatever may be its nature, which powerfully excites the emotions, whether the emotional disturbance be in the form of grief, fright, a fit of rage, great anxiety, or disappointment may bring on an attack. So also may causes or influences of a depressing character which tend to exhaust nerve force, whether from excessive bodily or mental exercise, or both. So also may the disease be induced as a result of reflex action consequent upon some local irritation. Nor do I think it necessary that in order that the disease be developed, the morbid local disturbance must be of a particular character, nor that it be located in any particular part of the body; it *may* be in the ovaries or uterus, it is *frequently* some where else. Why not? To claim that in every case of this malady the muscular movements and morbid manifestations have their focus in the reproductive system, would be doing violence, it seems to me, to the great law of reflex action. Why not the source of irritation be seated in some other part of the organism, as in bowels loaded with flatus or feculant matter? in a disordered stomach? a carious tooth or an inflammed eye? and so on *ad infinitum.* Again, how are we to reconcile the theory that insists that the cause of all hysterical symptoms is to be sought for in the sexual system, with the acknowledged fact that in many cases the most searching and rigid scrutiny utterly fails to detect in the sexual organs even the slightest derangement. In these cases how are we to escape the conclusion that the hysterical phenomena are due to a purely psychical cause? In other words, that the exciting cause is not peripheral, and therefore not uterine, but that it is primarily centric in its origin? Called to a case of this disease, it is improbable that any intelligent physician, with his wits well in hand, would be puzzled very long as to what the trouble is. There will be present in every case certain distinctive features which will generally enable him to arrive at a correct diagnosis with little difficulty. It should be remembered, however, that hysteria may be associated with other diseases, especially is this likely to be the case with individuals who possess the "hysterical diathesis."

A young medical friend of mine had under treatment, in the person of a young lady, a case of typhoid fever of mild type. For years she had been subject to hysterical attacks, which were almost certain to supervene on any physical ailment. At the very commencement of her illness hysterical symptoms developed themselves and soon became prominent. While the physician was resting easy and congratulating himself on the supposed fact that the case was one comparatively free from danger, his patient died. As to the prognosis in any given case of this disease, if we would be governed by its past history, we can always calculate with absolute certainty on a favorable result, or rather, I would say, that in no case of uncomplicated hysteria is there danger to life, for while many cases are cured, at least temporarily, there are many others that no method of treatment, medical or moral, can do anything for, but these latter never succumb to the disease. As regards the pathology of hysteria, the profession is groping its way in a labyrinth of doubt and uncertainty.

That it is a disease of the nervous system, characterized by exaggerated emotional disturbance, with impaired volition or will-power, is a proposition to which all, perhaps, will agree; but as to the primary seat of the disorder, whether its starting point is in the uterus, or whether it is to be found in the brain, spinal cord or ganglionic centers, is a question upon which there is a radical difference of opinion. The weight of authority, however, at present seems to favor the cerebral theory. In conclusion, a few words relating to the treatment of hysteria. This, to be at all successful, for obvious reasons, must necessarily embrace the medical, moral and hygienic. Of the many drugs which, from time to time, have been used in the treatment of the disease, it would probably be safe to say that nineteen-twentieths of them have proved worse than useless. There is perhaps no class of medicines more largely employed in this affection than the anti-spasmodics. It has fallen to my lot to treat many cases of hysteria; not a few of them representing every conceivable phase, grade and character of this wonderfully strange disorder; in common with many of my professional brethren, and in obedience to the teachings of many of our text books, I formerly depended, in a measure, for the relief and cure of my patients, upon the use of the reputed remedies just referred to. I have tried honestly and faithfully, with my limited professional knowledge and meager ability, to watch and note their effects, and I trust I will be pardoned if I give it as my deliberate opinion that, as remedial agents in hysteria, they are comparatively valueless. I have over and over again, in the treatment of the disease employed many of the so-called nervous stimulants or anti-spasmodics; I have used them in large doses and in small ones, at short intervals and at long intervals, and for days and weeks in succession; I have given valerian, which has been regarded by Dr. Meigs and others as almost a specific in this affection; I have given it freely and persistently, in powder, solid and fluid extract; I have used lactucarium, assafœtida, hyosciamus, musk, belladonna, valerianate of ammonia, sulph. ether, camphor, and I hardly know what else, and I cannot not now recall to mind a single instance in which the administration of any of these drugs, alone or in combination, was followed by any *marked* beneficial results. There *is* a medicine, however, which may almost *always* be relied upon. I refer to the bromide of potassium. For the purpose of calming and quieting the irritable condition of the nervous system, preventing convulsive seizures, exerting a favorable influence over the mental state and of *curing*, in many cases, the exaggerated sensibility, or hyperæsthesia, which not unfrequently exists, I know of nothing that will compare with it. I generally give it four or five times daily in from 20 to 30 grain (1–2 gm.) doses, and continue it, if necessary, for an indefinite period. To control the convulsions, which, to say the least of them, are always the source, to the physician, of no little annoyance, many rely on the use of cold water applied to the head after the method of the douche; this to my certain knowledge, in many cases, is highly efficacious. To succeed, however, it should be used copiously and freely, and persisted in until consciousness is fully restored and the patient begs for quarter. But if any doubts should be entertained in any given case, as to the propriety of applying the douche, chloroform or ether may be administered by inhalation. The cold douche may, and sometimes does, fail us, but I think I may safely say that either of these anæsthetics, given for the control of hysterical paroxysms, can be depended upon in every case, with absolute certainty. For the relief of the paralysis, which in a certain proportion of cases, is a prominent feature, I have used with good effect, strychnia, in connection with phosphorus; conjoined with these, high authority recommends the employment of electricity, by the primary or induced currents, or both, as may be thought best. In the paroxysms of coma, which now and then resist, at least for the time being, all forms of treatment, the douche not unfrequently works wonders; some resort to the procedure known as "firing" with satisfactory results; others again depend upon blistering. An example of what this latter method of counter-irritation will sometimes accomplish, has already been referred to in another part of this paper. Of course it will be admitted that the good accomplished by these measures, that is, the employment of the cold douche, the anæsthetics, the counter-irritation, or the galvanic current, is mainly, if not altogether, due to their moral effect. With the view of destroying as nearly as may be what has been very properly termed the "hysterical condition," I have been using for a number of years, with encouraging results, phosphorus, combined with some one of the preparations of strychnia, I usually employ the formula, first recommended, I think, by Dr. William A. Hammond, viz: nux vomica grs. x, phosphide of zinc grs. iij. Mix and divide into 30 pills; one pill three times daily before meals. In giving the above prescription I have found it necessary, in some cases, to lessen the proportion of phosphide of zinc at least one-fourth in consequence of its tendency to gastric disturbances.

To succeed with it however, it will be found necessary in not a few cases, to continue its use, for weeks or months. To relieve indigestion and other forms of gastric derangement so common in this disease, I have found the comp. tinc. of gentian to act well in many cases; and as there is not unfrequently more or less vomiting, I am in the habit of combining with the gentian the dil. hydrocyanic acid. I give of this prescription two drachms three times every day, awhile before each meal. In addition to the above or as a substitute therefor, should there be acid eructations, attended with a flatulent condition of the stomach and bowels, a very valuable remedy will be found in the sub. nit. or sub. carb. of bismuth, given in fifteen or twenty grain doses *after* meals. The hygienic management of the disease will, of course, very naturally address itself to the attention and good sense of every intelligent physician, hence I feel that anything I might be able to say in that connection would be not only unnecessary but superfluous.

But, gentlemen, in spite of our best directed efforts looking to the cure of this disease, regardless of any or all of the means which may be used, or methods adopted, a very large proportion of the cases remain uncured; and until science shall have advanced still further into the domain of medicine, revealing to us truths connected with its pathology and etiology of which we are at present ignorant, you will, I think, agree with me in the proposition that the medical treatment of hysteria must necessarily be, to a large extent, empirical.

